# Woman With Epigastric Pain

**DOI:** 10.1016/j.acepjo.2025.100144

**Published:** 2025-04-21

**Authors:** Meng-Hsi Chia, Hao-Cho Ou

**Affiliations:** Department of Emergency Medicine, Tri-Service General Hospital, National Defense Medical Center, Taipei, Taiwan

**Keywords:** epigastric pain, foreign body ingestion, ultrasonography of the abdomen, ultrasonography detection of foreign body

## 1 Patient Presentation

**Figure 1:**
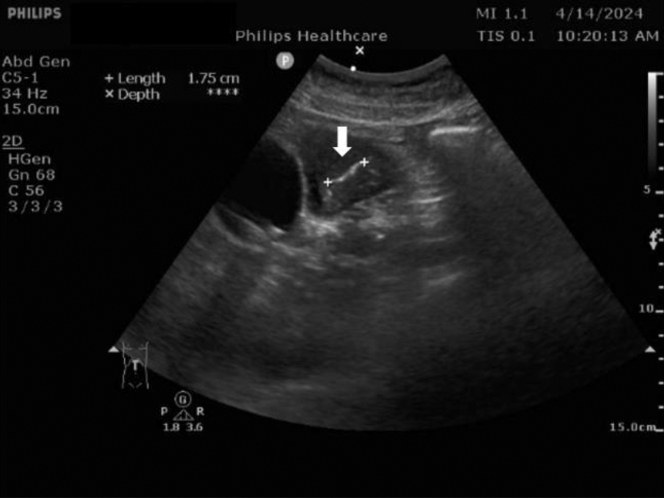
Bedside point-of-care ultrasonography showing a hyperechoic structure with acoustic shadow (arrowhead) over the stomach.

**Figure 2:**
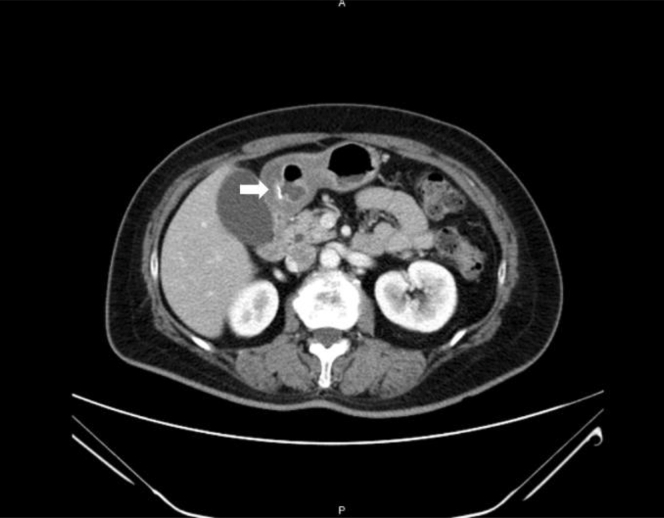
Computed tomography image of the abdomen reveals a hyperdense linear foreign body (arrowhead) in the gastric antrum.

A 67-year-old female with a history of Parkinson disease, hypertension, and diabetes mellitus under regular medical control presented to our emergency department due to epigastric pain for 3 days without fever. Physical examination revealed epigastric tenderness without Murphy’s sign. The laboratory examination results were normal. Chest radiography was negative. Bedside point-of-care ultrasonography of the abdomen (Fig 1) incidentally demonstrated a 1.75 cm hyperechoic linear structure within the gastric wall. A computed tomography was performed (Fig 2).

## 2 Diagnosis: An Ingested Foreign Body Lodged in the Antrum of the Stomach

**Figure 3:**
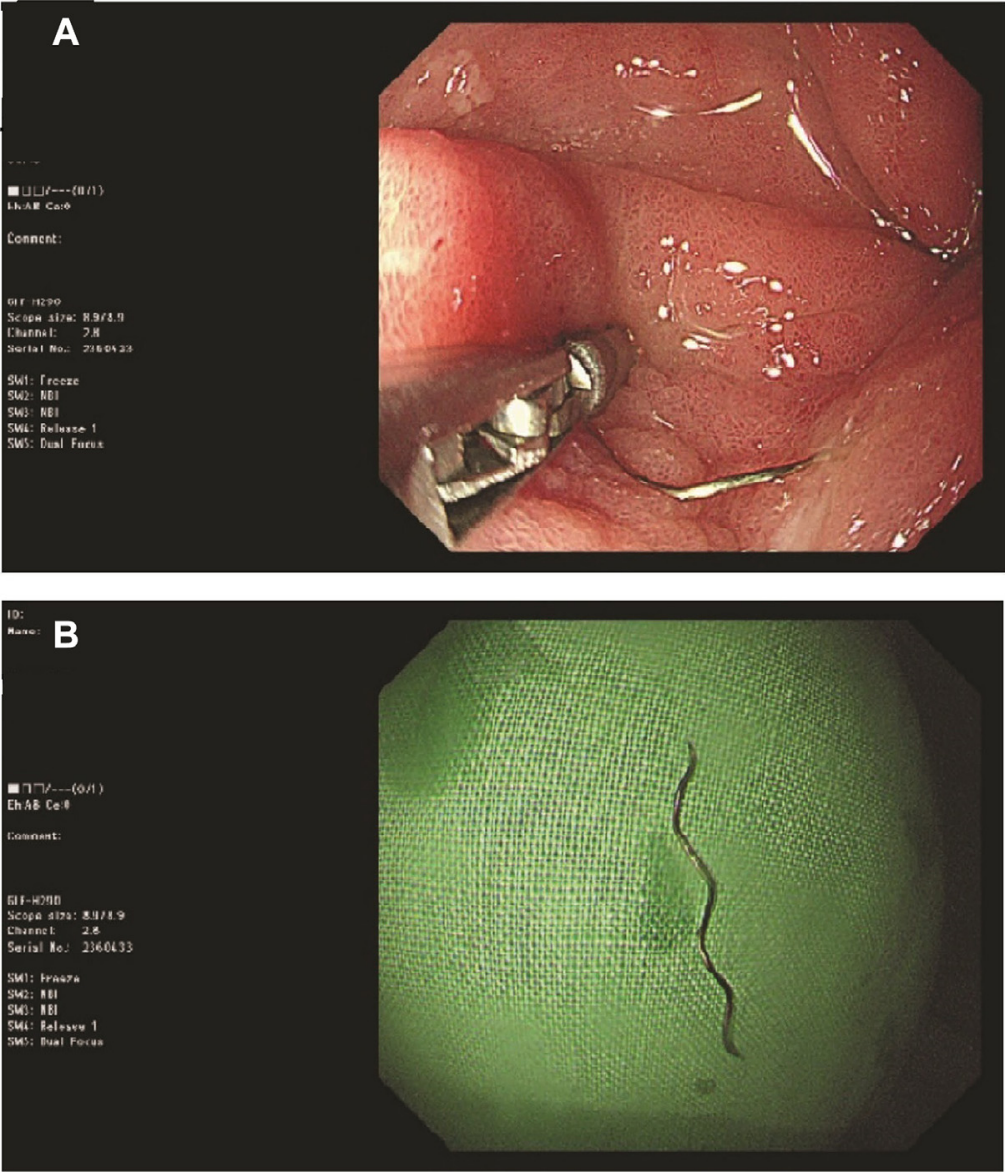
A, Upper gastrointestinal gastroscopy revealing erythematous change of gastric mucosa with a metal needle inserted in the antrum. B, The metal needle after removal.

Accidental foreign body ingestion is commonly observed in the emergency department. Most small ingested objects spontaneously pass through the gastrointestinal tract.^1^ However, serious complications, such as bowel perforation or obstruction, can occur.^2^ Ultrasonography may provide detailed information about the size, structure, and location of the foreign body, as well as the depth of its location. computed tomography may be a sensitive tool for detecting foreign bodies and foreign body-related complications.^3^ The patient underwent upper gastrointestinal gastroscopy (Fig 3A), during which the metal needle was removed (Fig 3B), and was discharged 3 days later.

## Funding and Support

No financial support to declare.

## Conflict of Interest

All authors have affirmed they have no conflicts of interest to declare.
